# Exercise and Mitochondrial Dynamics: Keeping in Shape with ROS and AMPK

**DOI:** 10.3390/antiox7010007

**Published:** 2018-01-06

**Authors:** Adam J. Trewin, Brandon J. Berry, Andrew P. Wojtovich

**Affiliations:** 1 Departments of Anesthesiology and Perioperative Medicine, University of Rochester Medical Center; Rochester, NY 14642 , USA; adam_trewin@urmc.rochester.edu; 2 Pharmacology and Physiology, University of Rochester Medical Center, Rochester, NY 14642, USA; Brandon_Berry@urmc.rochester.edu

**Keywords:** exercise, mitochondria, dynamics, energetics, reactive oxygen species, redox signaling, oxidative stress

## Abstract

Exercise is a robust stimulus for mitochondrial adaptations in skeletal muscle which consequently plays a central role in enhancing metabolic health. Despite this, the precise molecular events that underpin these beneficial effects remain elusive. In this review, we discuss molecular signals generated during exercise leading to altered mitochondrial morphology and dynamics. In particular, we focus on the interdependence between reactive oxygen species (ROS) and redox homeostasis, the sensing of cellular bioenergetic status via 5’ adenosine monophosphate (AMP)-activated protein kinase (AMPK), and the regulation of mitochondrial fission and fusion. Precisely how exercise regulates the network of these responses and their effects on mitochondrial dynamics is not fully understood at present. We highlight the limitations that exist with the techniques currently available, and discuss novel molecular tools to potentially advance the fields of redox biology and mitochondrial bioenergetics. Ultimately, a greater understanding of these processes may lead to novel mitochondria-targeted therapeutic strategies to augment or mimic exercise in order to attenuate or reverse pathophysiology.

## 1. Introduction

Exercise is a front-line intervention for the prevention and treatment of a large range of chronic diseases, including obesity, type-2 diabetes, metabolic syndrome, neurological disease, osteoporosis, and cardiovascular disease [1,2,3,4]. While the overall benefits of exercise on health outcomes are unequivocal, there is poor compliance to the recommended physical activity levels at the population level worldwide [5,6]. Therefore, much attention has been paid to developing ways to augment or mimic exercise responses [7,8], which could benefit people who are unwilling or unable to meet recommended physical activity levels. Despite this, whether a bona fide exercise mimetic (e.g., exercise in a pill) is at all possible has been questioned, mainly because of the vast range of effects that exercise has on all levels of physiology [9,10]. Nevertheless, many therapeutic targets are yet to be discovered since the molecular mechanisms that underlie the beneficial effects of exercise remain incompletely understood [11,12].

Skeletal muscle is a key endocrine and metabolic organ contributing to 30% of whole body metabolism at rest, and up to 90% during maximal exercise [13]. Indeed, mitochondrial ATP synthesis in skeletal muscle is critical to maintain energy homeostasis during the ~100-fold increase in bioenergetic demand that occurs during exercise [14]. Mitochondrial adaptations involving an increased synthesis of mitochondrial electron transport system (ETS) proteins (i.e., mitochondrial biogenesis) following exercise training are therefore linked to improved metabolic health [15,16]. Discussions of mitochondrial function most commonly refer to respiratory activity of the ETS and the resulting synthesis of ATP via oxidative phosphorylation (OXPHOS). However, intrinsically linked to respiratory activity is the generation of reactive oxygen species (ROS), which is currently thought to occur from at least 11 sites within the ETS and its associated peripheral proteins [17]. The varying rates of ROS generation due to fluctuating bioenergetic conditions play a vital and interdependent role in overall mitochondrial function [18]. Furthermore, mitochondrial bioenergetics and ROS generation are influenced by, and influence, the morphology and dynamics of mitochondria [19,20,21]. In skeletal muscle, mitochondrial morphology is a highly connected reticulum that allows the distribution of substrates and products to areas of the greatest bioenergetic demand [22,23]. Therefore, it is reasonable to consider how the cellular perturbations imposed by exercise stimuli affect mitochondrial dynamics and function. For instance, it is known that exercise leads to increased mitochondrial mass (i.e., biogenesis) and the upregulation of cellular antioxidant defense systems, and that exogenous non-specific antioxidant supplementation often impairs these adaptive responses. Despite this, the acute effects of exercise on early molecular responses which regulate intrinsic mitochondrial function and dynamics remain relatively less understood. In this review, we will discuss the interdependent molecular mechanisms between mitochondrial dynamics, bioenergetics, and redox homeostasis, with respect to how an exercise stimulus may act upon this network to orchestrate beneficial effects on (patho)physiology.

## 2. Mitochondrial Morphology and Dynamics

**Figure 1:**
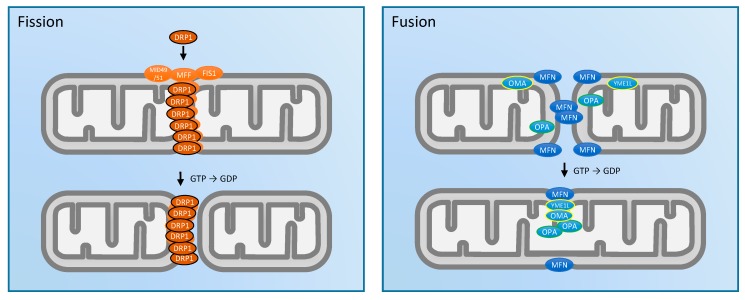
Overview of key proteins involved in mitochondrial fission and fusion. Fission processes depicted in orange: dynamin-related protein-1 (DRP1) can bind to a range of receptor proteins mitochondrial fission factor (MFF), mitochondrial fission 1 protein (FIS1), and mitochondrial dynamics proteins of 49 and 51 kDa (MID49/51) on the outer mitochondrial membrane. Upon guanosine triphosphate (GTP) hydrolysis, DRP1 oligomers constrict to divide mitochondria into separate organelles. Fusion processes depicted in blue: GTPase mitofusin (MFN1/2) of separate mitochondria dimerize, and then pull together upon GTP hydrolysis to fuse the outer mitochondrial membranes (OMM). The inner mitochondrial membrane (IMM) is fused by the binding of optic atrophy-1 (OPA1) which faces the intermembrane space (IMS) while tethered to the IMM of each incoming mitochondria. Regulation of IMM fusion occurs via proteases metalloendopeptidase mitochondrial (OMA) and ATP-dependent zinc metalloprotease 1 (YME1L) which cleave the membrane tethered domain of OPA1 from the IMM, rendering it non-functional. GDP: guanosine diphosphate.

Mitochondria are dynamic organelles that constantly move throughout the cell in order to mix mtDNA and protein contents and to remove damaged components. Mitochondria are also trafficked to different regions in the cell where energy is needed. This requires changes in mitochondrial movement throughout the cell (i.e., active transport) and morphology via the delicate balance between the fission (separation) and fusion (joining) of mitochondrial membranes. These processes are performed by specialized protein machinery (reviewed extensively in [24,25,26]) at the outer and inner mitochondrial membranes (OMM and IMM, respectively). As summarized in Figure 1, fission is actuated by dynamin-related protein-1 (DRP1), a molecular motor protein that binds to the OMM receptors mitochondrial fission 1 protein (FIS1), mitochondrial fission factor (MFF), and mitochondrial dynamics proteins of 49 and 51 kDa (MID51/49), leading to oligomerization as a ring around a mitochondrion [27]. Notably, MFF binds DRP1 with a higher affinity than FIS1 in mammalian cells [28]. Upon guanosine triphosphate (GTP) hydrolysis, the DRP1 ring constricts around a mitochondrion, separating it into two or more discreet organelles [29]. Fission may then facilitate mitophagy, a mitochondria-specific autophagy process which marks damaged regions for lysosomal degradation [30,31].

Conversely, to achieve membrane fusion, dimerization of the GTPase mitofusin (MFN1/2) occurs at the OMM between two or more separate mitochondria, which are drawn together upon GTP hydrolysis [26,32]. Once MFN1/2 fuses the OMM of adjacent mitochondria, another GTPase optic atrophy-1 (OPA1) fuses the IMM compartments [33]. OPA1 in its non-processed long form (L-OPA1) is tethered to the inner membrane facing the intermembrane space (IMS), allowing it to bind another L-OPA1 on the IMM of the incoming mitochondrion. This process is tightly regulated under basal conditions, with about half of L-OPA1 degraded by ATP-dependent zinc metalloprotease 1 (YME1L) and metalloendopeptidase mitochondrial 1 (OMA1) into an inactive IMS soluble short form (S-OPA1) [34,35]. Overall, these fission/fusion events can occur extremely rapidly—often within a matter of seconds [36].

Various scaffolding proteins are also involved in structure and morphology within and between mitochondria, which are functionally distinct from fission/fusion proteins. For instance, intermitochondrial junctions (IMJ) are formed by an OMM protein, CDGSH iron-sulfur domain-containing protein 1 (also known as mitoNEET, based on its amino acid sequence), which structurally tethers adjacent mitochondria without actually fusing [37,38]. Within the IMS, large protein complexes exist, known as the mitochondrial contact site and cristae organizing system (MICOS) [39]. Currently, nine subunits and interactors have been identified in mammalian cells as constituents of MICOS and are named MIC10 to MIC60 based on molecular weight [40]. This includes proteins otherwise known as mitofilin (MIC60) [41] and a recently discovered MIC13 (also known as QIL1) [42], which collectively function to tether the OMM to the IMM at cristae junctions (CJ). At the CJ, MICOS also interacts with cardiolipin, a phospholipid in the IMM which helps give cristae its curvature [43,44]. The resulting cristae-fold ultrastructure allows for the distribution of OXPHOS proteins within specific cristae microdomains [45]. This appears to be important for the formation of respiratory supercomplexes (i.e., clusters of ETS complexes) which enhance ETS/OXPHOS activity by decreasing the diffusion distances of substrates and electron transfer [20]. Also necessary to maintain CJ integrity is the redox sensitive protein reactive oxygen species modulator 1 (ROMO1), which has also been shown to interact with OPA1 [46]. 

A final aspect of mitochondrial dynamics is their active transport throughout the cell to regions of energetic demand. This is performed by mitochondrial Rho GTPase and trafficking kinesin-binding protein (MIRO/TRAK) complexes which link the mitochondrial OMM adapter protein MIRO to kinesin motor proteins which “walk” along microtubules and microfilaments of the cytoskeleton [47]. In certain cell types such as skeletal muscle where intermyofibrillar mitochondria have more restricted motility due to the ultrastructure of the surrounding contractile machinery, these active transport proteins are also involved in forming protrusions that sprout from immobilized mitochondria known as nanotunnels [48,49].

Overall, the importance of mitochondrial dynamics to cellular physiology is highlighted by the severe detrimental effects to mitochondrial function in the absence of most of these key proteins [50,51]. The expression of these proteins and the processes which modulate their function are dynamically regulated at multiple levels by various cellular signals. In the following sections, we discuss emerging evidence for the roles of redox and bioenergetic homeostasis as key regulators of mitochondrial morphology and dynamics.

## 3. Mitochondrial Dynamics and ROS

Mitochondrial dynamics and morphology have increasingly been shown to be regulated by reactive oxygen species (ROS) and reactive nitrogen species (RNS). ROS/RNS are central to redox homeostasis—the balance between reduction and oxidation reactions via the gain or loss of electrons. Numerous cytosolic enzymes generate superoxide anion (O_2_**•**^−^), such as nicotinamide adenine dinucleotide phosphate (NADPH) oxidases, xanthine oxidase, monoamine oxidases, and phospholipases [52]; while the primary form of RNS, nitric oxide, is generated by nitric oxide synthases (NOS) [52]. In addition, the mitochondrial ETS generates O_2_**•**^−^ from at least 11 different sites, at varying rates depending on the respiratory state [17,53]. Dismutation of O_2_**•**^−^ occurs either spontaneously in a first-order reaction, or enzymatically by superoxide dismutase (SOD) to hydrogen peroxide (H_2_O_2_), which can be scavenged by reduced glutathione (GSH), resulting in oxidized glutathione (GSSG), and by enzymes including glutathione peroxidase (GPX), thioredoxin/peroxiredoxin (TRX and PRDX), and catalase [54].

**Figure 2:**
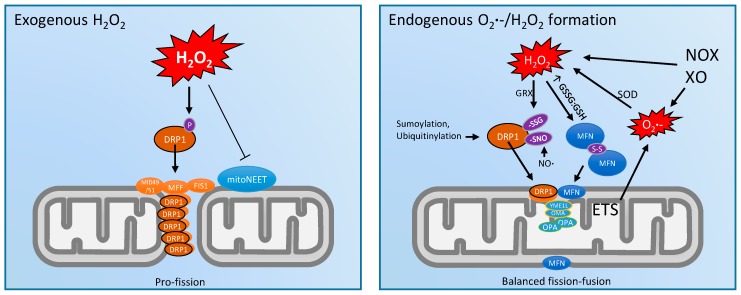
Regulatory responses of mitochondrial dynamics machinery to exogenous vs. endogenous reactive oxygen species (ROS) in the form of superoxide (O_2_**•**^−^) and/or hydrogen peroxide (H_2_O_2_). Exogenous H_2_O_2_ application (often used experimentally at supraphysiologic concentrations) leads to fragmentation via the activation of DRP1 via phosphorylation at Ser616 and also a mitoNEET dependent mechanism. Endogenous ROS generated in specific microdomains such as sites within the electron transport system (ETS), NADPH oxidase (NOX) or xanthine oxidase (XO) enzymes target numerous redox active cysteine residues contained within both fission and fusion proteins via *S*-glutathionylation (protein–SSG), disulfide bond formation (S–S), and *S*-nitrosation (protein–SNO) post-translational modifications. This allows precise control of mitochondrial dynamics in response to spatial and temporal changes in ROS. GSSG: oxidized glutathione; GSH: reduced glutathione; GRX: glutaredoxin; SOD: superoxide dismutase.

Several studies have monitored mitochondrial dynamics in cell models in response to exogenous H_2_O_2_ (Figure 2). High concentrations (3–16 mM) of exogenous H_2_O_2_ were shown to induce dose dependent mitochondrial fragmentation in human umbilical vein endothelial cells, and also to increase the expression of a number of both fission and fusion genes [55]. Similarly, the incubation of C2C12 myocytes (a commonly used skeletal muscle model) at a lower concentration (250 µM) of exogenous H_2_O_2_ for 1–25 h led to a time-dependent fragmentation of the mitochondrial reticulum [56], although *mfn1/2*, *opa1*, *dnml1*, and *fis1* mRNA expression were unaffected [56]. The mechanism(s) for exogenous H_2_O_2_ induced mitochondrial fragmentation in C2C12 myocytes were later shown to involve increased DRP1 activity [57]. In addition, it was recently shown that mitochondria with a mutation in an iron-sulfur (Fe-S) cluster in mitoNEET were resistant to H_2_O_2_ induced fragmentation [37]. This is interesting given that Fe-S clusters are typically thought to be oxidized by O_2_**•**^−^, not H_2_O_2_. Finally, mitochondrial transport dynamics were recently shown to be decreased acutely and reversibly by exogenous H_2_O_2_ and intracellular oxidant generation [58]. 

Regarding how these processes are regulated at the molecular level, there is emerging evidence for the redox regulation of fission/fusion proteins via post-translational modifications (PTMs) [59]. For instance, in addition to the well characterized regulation of DRP1 by phosphorylation at serine residues 616 and 637 [60], ubiquitination, and sumoylation [25], DRP1 and its interactor proteins also contain cysteine residues, making them susceptible to redox-mediated PTMs such as S-glutathionylation and S-nitrosation [59,61,62]. Additionally, a key mechanism for OMM fusion between adjacent mitochondria involves dimerization of MFN1/2 via disulfide bond formation at Cys684 in response to elevated levels of oxidized glutathione (GSSG) [63,64]. Although this pro-fusion response to elevated GSSG is seemingly at odds with the pro-fission response to exogenous H_2_O_2_, it is probably difficult to compare the relatively blunt effects of exogenous vs. spatially and temporally confined endogenous redox perturbations in vivo.

At the transcriptional level, H_2_O_2_ acts as a second-messenger signaling molecule [65] and may influence the expression of mitochondrial dynamics genes. For instance, the oxidative stress sensing transcription factor nuclear factor erythroid 2-related factor 2 (commonly referred to as NRF2), is a master regulator of the expression of numerous stress resistance and antioxidant target genes [66]. However, NRF2 activation has also recently been shown to promote mitochondrial fusion by increasing the expression of components of the 20S proteasome that led to the enhanced ubiquitin-independent degradation of DRP1 [67]. In addition, the peroxisome proliferator-activated receptor gamma coactivator (PGC1α/β) signaling pathway, central to the mitochondrial biogenesis transcriptional program, is known to be redox sensitive [68], which may be pertinent since PGC1β has also been shown to regulate MFN2 expression [69]. Taken together, redox reactions have complex effects on mitochondrial dynamics at multiple levels of regulation.

Given the complexity of these regulatory processes, it seems likely that the reverse relationship could also exist—i.e., that manipulation of mitochondrial dynamics can directly alter ROS generation. Computational simulation has provided interesting insight into how mitochondrial spatial distribution can have substantial effects on the degree of spatial and temporal ROS signal propagation [70], perhaps as a function of feed-forward ROS induced ROS Release (RIRR) [71]. Consistent with this notion, mitofilin, part of the intermembrane tethering MICOS complex, was shown to be required for redox homeostasis both in cells [72] and *C. elegans* [41]. Moreover, Mdivi1, a quinazolinone derivative identified as a DRP1 inhibitor [73], was shown to decrease ROS formation in response to nutrient overload stress [74]. This suggests that mitochondrial fusion decreases the propensity for mitochondrial ROS generation or release. It should be noted that the specificity of Mdivi1 for DRP1 has recently been questioned due to inhibitory effects on complex-I activity [75], although the overall effects of Mdivi1 inhibition of DRP1 to decrease ROS formation are consistent with genetic approaches using an inactive DRP1 variant [76]. Careful consideration should be given, however, when assessing mitochondrial dynamics following the deletion or knockdown of a single gene of the fission/fusion machinery due to possible compensatory responses and/or epistasis [77].

## 4. Mitochondrial Dynamics and 5'-Adenosine Monophosphate (AMP)-Activated Protein Kinase (AMPK): Regulation by ROS?

**Figure 3:**
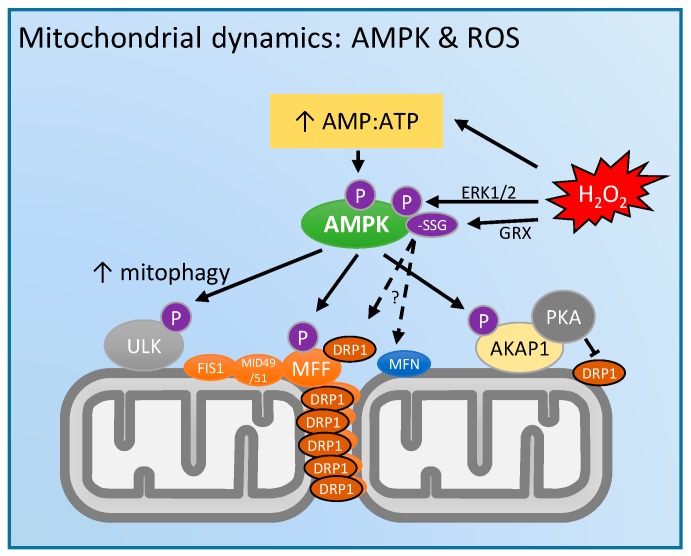
Known and putative roles of 5'-adenosine monophosphate (AMP)-activated protein kinase (AMPK) and ROS mediated regulation of mitochondrial dynamics processes. Under energetically stressful conditions, rising AMP levels relative to ATP are sensed by AMPK which leads to the phosphorylation of downstream targets including: MFF to promote DRP1 binding, unc-51 like autophagy activating kinase (ULK) to induce mitophagy, and A-kinase anchoring protein mitochondrial (AKAP1) to bind cyclic-AMP-dependent protein kinase (PKA), leading to the inhibitory phosphorylation of DRP1 Ser637. In addition, ROS may modulate AMPK via AMP:ATP levels, extracellular signal-regulated kinase (ERK1/2) mediated phosphorylation, and additionally via glutaredoxin (GRX) mediated *S*-glutathionylation. However, the redox regulation of AMPK has not been experimentally shown to directly modulate fission/fusion dynamics.

Mitochondrial dynamics and bioenergetics display an interdependent relationship: changes in mitochondrial network connectivity alter OXPHOS efficiency and function, and vice-versa. The mechanistic links between sensing cellular energy status and mitochondrial dynamics are increasingly being thought to involve AMPK (Figure 3). AMPK is a key cytosolic metabolic sensor comprised of two regulatory β and γ subunits and a catalytic α subunit. In conditions of high energy turnover when adenylate kinase is unable to prevent elevation of the AMP:ATP ratio, AMP allosterically regulates AMPK activity, along with PTMs at a number of key residues [78]. Interestingly, Toyama et al. [79] recently showed that impaired mitochondrial bioenergetics (induced via rotenone and/or antimycin-A treatment to inhibit ETS complexes I and III, respectively) induced AMPK activation as expected, but that this AMPK response was also necessary for mitochondrial fission. Notably, their study identified MFF, the key OMM receptor protein for DRP1, as an AMPK substrate, and that the phosphorylation of MFF by AMPK was necessary for fission. Further, they showed that pharmacologic activation of AMPK alone is sufficient to induce mitochondrial fission, consistent with an earlier investigation [80]. It was also recently shown that AMPK is both necessary and sufficient for unc-51 like autophagy activating kinase 1 (ULK1) phosphorylation to initiate mitophagy and mitochondrial transport to lysosomes [81]. Together, these studies demonstrate that AMPK activation is a key regulator of mitochondrial fission and the functional significance of this may be to initiate the mitophagy of damaged mitochondrial regions.

There is mounting evidence that AMPK activity is redox sensitive [78]. It was originally shown by Choi et al. that the exposure of 100–600 µM exogenous H_2_O_2_ increased AMPK activity due to an H_2_O_2_ induced increase in the AMP:ATP ratio [82]. They also reported a separate mechanism involving extracellular regulated kinase (ERK1/2) mediated phosphorylation of the critical Thr172 residue in the catalytic α-subunit of AMPK. Later, using AMP-insensitive mutant cells, Zmijewski et al. demonstrated that AMPK activity was increased by *S*-glutathionylation of Cys 299 and 304 in the α subunit, which preceded increases in AMP:ATP [83]. Although Hawley et al. [84] initially questioned this report, they were later able to recapitulate the earlier finding when AMPK activity was measured within a shorter timeframe after H_2_O_2_ exposure (10 min vs. 1 h) [85]. Shao et al. showed that the oxidation of other Cys residues (130 and 174) in the α subunit can induce the aggregation of AMPK, thus blocking its ability to be phosphorylated by its upstream kinases [86], highlighting the importance of maintaining redox homeostasis for canonical AMPK activity. Consistent with this, Dong et al. recently showed that elevated GSSG levels inhibited glutaredoxin (GRX) mediated *S*-glutathionylation of AMPK necessary to increase its activity in response to 15 min exposure to 10 µM H_2_O_2_ [87]. 

Collectively, these studies show that ROS can regulate AMPK activity, and that these rapid regulatory responses may have an important functional significance to maintaining mitochondrial function in the face of acute cellular stress. However, the hypothesized control of mitochondrial dynamics specifically via the redox regulation of AMPK has yet to be demonstrated experimentally.

## 5. Exercise Regulation of Mitochondrial Dynamics: Via ROS and/or AMPK?

Exercise generally involves repeated contractions of one or more groups of skeletal muscles. This can be for prolonged periods of time (i.e., multiple hours) at low-moderate intensity (e.g., aerobic exercise), and/or for increasingly shorter and more intense bouts such as high intensity interval exercise (HIIE), sprint (typically <30 s duration). Additionally, resistance exercise consists of high force contraction in both concentric (shortening under tension) and eccentric (lengthening under tension) contraction characteristics. Although these different modes of exercise have distinct bioenergetic requirements, skeletal muscle mitochondrial function is undoubtedly fundamental to bioenergetics in all of these conditions, as evidenced by the robust induction of mitochondrial biogenesis transcriptional responses with each of these [15,88,89]. However, a greater understanding of how both acute exercise (e.g., within a few hours post-exercise), as well as repeated bouts of exercise (i.e., training), is related to mitochondrial dynamics is necessary.

Seminal studies using transmission electron microscopy in the late 1960s onwards by Penman [90], Gollnick [91,92], Kiessling [93], Kirkwood [94], and others revealed that exercise induces morphological changes to mitochondria in skeletal muscle both in rodents and humans. These studies collectively show that the changes in response to acute exercise involve mitochondrial swelling and/or fragmentation, but after recovery and/or with exercise training, there is increased mitochondrial volume and connectivity. More recent studies have focused on measuring the expression of fission/fusion genes and proteins in response to single sessions of exercise (Table 1), as well as repeated bouts of exercise training (Table 2).

For instance, it was first shown by Cartoni et al. [95] that *mfn1/2* mRNA expression is unchanged 2 h after a single intense exercise bout, but increased at 24 h post exercise. This was shown to be mediated by two critical exercise-responsive and transcriptional co-activators PGC1α and estrogen-related receptor (ERRα), which drive mitochondrial biogenesis; however, no resulting changes in the protein level of MFN2 were observed at the time points assessed [95]. Subsequently, Perry et al. [96] reported unchanged MFN1/2, FIS1, and DRP1 protein 4 h after a single session of high intensity interval exercise (HIIE) in humans, but after two weeks of this HIIE training, MFN1, FIS1, and DRP1 protein content were increased. A number of more recent studies [57,97,98,99] have also shown similar skeletal muscle MFN1/2 increases with training, but not all [100,101]. These variable responses may be explained by differences in the nature of the exercise stimulus, prior training status [102], species, sex, age, nutrition status (including dietary antioxidant intake), and muscle fiber-type, as recently shown in older humans [103]. Collectively however, most studies seem to report some increase in the expression of fusion proteins in response to long-term exercise training (Table 2).

A limited number of studies have assessed in vivo responses to exercise under conditions of knockdown or overexpression of mitochondrial dynamics proteins. Using a haploinsufficient *Opa1* mouse model (~50% of wild-type OPA1 expression), Caffin et al. reported an enlarged mitochondrial ultrastructure with altered cristae morphology [109]. These mutant mice paradoxically displayed an increased exercise endurance capacity compared to wild-type individuals in response to training, which was apparently due to a compensatory increase in mitochondrial fatty acid transport and oxidation. Whether the effects of OPA1 deficiency were primary or compensatory to the observed exercise phenotype remains to be elucidated. Recently, Weir et al. showed that the deletion of either fission or fusion proteins was detrimental to *C. elegans* lifespan and mitochondrial morphology; whereas the loss of fission and fusion proteins (double knockout) was not [110]. Interestingly, while these double knockout animals had a relatively normal mitochondrial morphology and were phenotypically similar to wild-type animals under basal conditions, their mitochondrial morphology was unable to dynamically respond to energetic stress induced by intermittent fasting. This finding is consistent with responses in cardiac specific triple-knockout of Mfn1/Mfn2/Drp1 in mice, where the balanced loss of mitochondrial dynamics resulted in a less severe phenotype than the imbalanced loss of fission or fusion processes individually [111].

Another recent report by Coronado et al. showed that cardiac mitochondrial fragmentation is a physiologic response to acute exercise in mice [112]. When treated with the DRP1 inhibitors Mdivi1 or P110 (a peptide inhibitor of FIS1) immediately prior to exercise, mice displayed less exercise-induced mitochondrial fragmentation, which negatively affected their exercise capacity and impaired their ex vivo mitochondrial respiratory function. Together, these findings support the notion that mild mitochondrial fragmentation is a necessary response to increased bioenergetic flux during exercise.

**Figure 4:**
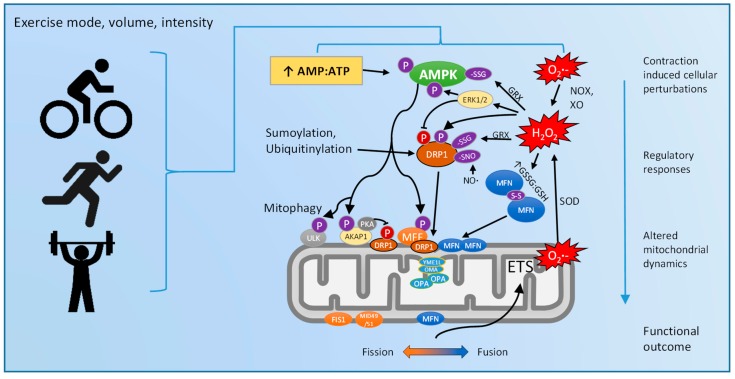
Proposed effects of exercise on mitochondrial dynamics via AMPK and ROS linked mechanisms. Exercise of distinct mode, volume, and intensity may have differential effects on cellular perturbations. This includes an increased bioenergetic demand resulting in an increased AMP:ATP ratio, along with increased contraction mediated and post-exercise ROS formation. These perturbations are sensed by AMPK, which initiates a cascade of phosphorylation signaling events that are interlinked with redox mediated post translational modifications. The specific activation or inhibition of each fission (depicted in orange) or fusion (depicted in blue) effector results in a net mitochondrial dynamics response which allows the myocyte to better meet the localized bioenergetic requirements of subsequent energetic stress.

While protein abundance is important for the overall capacity to undergo fission/fusion events, it is the acute regulatory events such as post-translational protein modifications which trigger the actual morphological changes observed. For instance, Picard et al. [105] used transmission electron microscopy to demonstrate that in mouse skeletal muscle following a single three hour session of “low” intensity voluntary treadmill running (to avoid mitochondrial swelling), both subsarcolemmal and intermyofibrillar localized mitochondria had an increased number of intermitochondrial contact points which are associated with subsequent membrane fusion, yet this occurred in the absence of overt changes to morphology or MFN2 or OPA1 protein abundance. To this end, relatively little is known about how exercise acutely regulates the function of the fission/fusion machinery (Figure 4).

Exercise acutely and markedly increases bioenergetic fluxes from ATP consumption from contractile (actin-myosin crossbridge cycling) and noncontractile processes [113]. In skeletal muscle, the mitochondrial reticulum is known to be highly connected and dynamically regulated by bioenergetic fluxes in order to optimally distribute substrates, ATP, and mitochondrial membrane potential [22,114,115]. Since mitochondrial fusion is known to be a membrane potential-dependent process [116,117], moderate decreases in mitochondrial membrane potential due to maximal energetic demand [118,119], such as during intense exercise, could shift the balance towards pro-fission. This may provide some explanation for “swollen” or fragmented mitochondria after acute bouts of intense exercise [91]. These acute post-exercise pro-fission responses in mitochondrial dynamics may be later surpassed by pro-fusion adaptive responses. Beneficial changes may include a more tightly packed cristae morphology [120] and ETS supercomplex assembly to increase the OXPHOS efficiency [121], which would subsequently allow for a better distribution of energy throughout the muscle cell [22].

Increases in bioenergetic demand during exercise result in an increased AMP:ATP ratio, which is sensed by AMPK [122,123,124]. Consistent with the work of Toyama et al. [79], Hoffman et al. reported that MFF is phosphorylated by AMPK after acute exercise [123], highlighting a pro-fission response to acute exercise. Another pro-fission response to acute exercise is likely via the increased stimulatory phosphorylation of DRP1 at Ser616 [97,106], possibly by the upstream kinase ERK1/2 which is known to be readily activated by exercise [125]. Interestingly, after training, basal DRP1 Ser616 phosphorylation levels are decreased [108]. Also in response to exercise, Hoffman et al. [123] demonstrated that AMPK can phosphorylate the OMM protein A-kinase anchoring protein (AKAP1) at Ser103. Upon its phosphorylation, AKAP1 binds protein kinase A (PKA) to the OMM, and the increased PKA activity resulting from exercise [123] phosphorylates DRP1 at Ser637. Phosphorylation at this site is inhibitory to its GTPase activity [126,127], thus overall exerting a pro-fusion effect in an AMPK dependent manner.

Furthermore, the recent interest in AMPK as a redox sensitive signaling hub has been considered in the context of exercise [128]. Indeed, exercise increases net ROS formation during exercise [129,130,131,132]. Increased ROS occurs mostly from non-mitochondrial sources during contractile activity such as NADPH oxidases and xanthine oxidase [17,133,134], and mitochondria are likely a primary source of O_2_•^−^ generation in the post exercise period [53]. Importantly, post-exercise mitochondrial O_2_•^−^ generation may be altered in a respiratory state-dependent manner [135]. This may be a result of exercise-induced post-translational modifications to mitochondrial ETS proteins and/or antioxidant enzymes [53,136,137,138]. These spatial and temporal changes in ROS formation and removal are important for appropriate responses to exercise [125,139]. Thus, based on cell-based experiments and correlative data, it seems reasonable to hypothesize that exercise-mediated alterations in ROS generation and redox homeostasis could regulate the redox components of mitochondrial dynamics. This could occur either via direct oxidative modifications to fission/fusion proteins, or indirectly via a redox sensitive AMPK-mediated mechanism.

In summary, there are separate lines of evidence largely from in vitro studies that demonstrate multiple levels of regulation of the complex relationships between (a) ROS and mitochondrial dynamics; (b) AMPK and mitochondrial dynamics; and (c) ROS and AMPK. However, to the best of our knowledge, no studies to date have systematically investigated the potential interdependent relationships between all three, either in vitro or in vivo. As a systemic physiologic stimulus, it seems likely that exercise could simultaneously target all of the aforementioned pathways. Perhaps it is a sum of all these and likely many other regulatory mechanisms whose interdependent relationships enable the fine tuning of the overall mitochondrial dynamics responses to exercise. A greater understanding of these mechanisms may pave the way toward novel therapeutic strategies. However, an experimental investigation of such a complex system presents a number of methodological challenges.

## 6. Future Research and Novel Methodologies

Experimental manipulation of ROS is fundamentally challenging given that their effects are highly dependent upon their spatial and temporal generation. Mitochondrial ETS inhibitors such as rotenone and antimycin-A lead to O_2_•^−^ generation at complex I and III. However, a major limitation is that the effects specific to oxidant generation are difficult to discriminate from the inhibition of mitochondrial function. Recently, Brand and colleagues have developed small molecules which bind with high specificity to complex I and III (known as Suppressors of site I_Q_ and III_Qo_ Electron Leak—S1QEL and S3QEL, respectively) [140,141]. These molecules reportedly suppress O_2_•^−^ generation at these sites without affecting respiratory activity. It will be interesting to investigate the role of post-exercise mitochondrial O_2_•^−^ mediated adaptive responses using these molecules, considering that many “global” antioxidant supplementation studies often impair beneficial exercise responses [125,134,142,143,144]. The effects of precise manipulation of site specific ROS formation on mitochondrial respiratory function may be assessed by high-throughput [145] or high-resolution respirometry methods [146]. Despite the significant advance that this represents to investigate mitochondrial ROS-specific effects on physiology, like any pharmacologic intervention, there exists a lack of ability to rapidly reverse the action. 

A novel approach to address this challenge is the utilization of optogenetics, which uses light to activate genetically encoded photosensitive proteins. To this end, an increasing range of photosensitive proteins has been developed such as selective ion channels [147,148] and pumps [149], as well as photoactivatable “caged” kinases [150]. Notably, optogenetic ROS generating proteins (RGPs) are available, such as KillerRed and SuperNova, which generate O_2_•^−^ upon illumination. This is achieved using a specific wavelength of light, allowing for reversible and precise temporal control of ROS generation [151]. Currently, it is unknown whether there are compartment specific effects of ROS in response to exercise. These optogenetic tools can be combined with the use of tissue specific promoters, optogenetic proteins can be expressed in specific cell-types in vivo, and/or be fused to an endogenous protein to achieve spatial control of oxidant generation in a specific cellular microdomain [152,153]. In support of this, it was shown very recently that KillerRed targeted to the mitochondrial matrix can induce acute mitochondrial fission in hippocampal cells [154]. Moreover, numerous genetically encoded fluorescent redox sensors are available to indicate compartment specific redox status in vivo [155]. 

Because light penetration in tissues such as skeletal muscle may pose limitations for some optogenetic studies, model organisms such as *C. elegans*, can be utilized due to their transparency and established methods for genetic manipulation [152]. Importantly, *C. elegans* have recently been shown to possess conserved mammalian molecular exercise responses relating to energy metabolism, mitochondrial redox homeostasis [156], and mitochondrial morphology [157]. Therefore, the collection of these relatively new tools offers unique opportunities to elucidate the regulatory links between ROS-AMPK-mitochondrial dynamics in response to exercise. Currently, there are many open questions including: what are the precise sources of ROS during and after exercise? How does ROS regulate mitochondrial dynamics in (patho)physiology? Are different tissues and/or subcellular populations of mitochondria differentially involved in the beneficial exercise phenotype, and are there distinct mechanisms regulating their dynamics? Does exercise regulate individual aspects of mitochondrial dynamics (i.e., biogenesis, fission, fusion, motility, and mitophagy) independent of each other, or are these responses “networked”? Answering these questions could have important implications not only for understanding the mechanisms that underlie the benefits of exercise, but also for the plethora of diseases associated with mitochondrial dysfunction [24,158].

## 7. Conclusions

**Figure 5:**
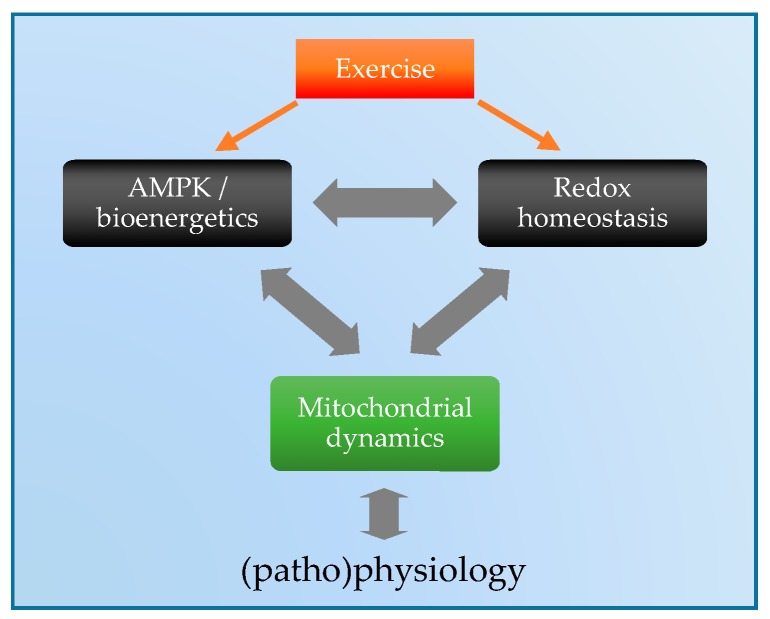
Proposed model for the interdependent regulation of mitochondrial dynamics in response to exercise.

In summary, mitochondrial dynamics are critical for organisms to adapt to various stressors; however, elucidation of the precise underlying molecular mechanisms which regulate these responses is currently incomplete. Exercise is a potent stressor to mitochondrial bioenergetics and redox homeostasis, and these signals may be sensed by AMPK to fine tune the regulation of mitochondrial dynamics (Figure 5). The development of new tools to accurately measure and generate ROS in a far greater spatial and temporal resolution within specific microdomains will be crucial to better understand these complex mechanisms in the context of exercise. Collectively, further research in this field could lead to the identification of novel therapeutic targets to augment or mimic key molecular aspects of exercise responses, which may have implications for numerous prevalent diseases.

## Figures and Tables

**Table 1:** Effects of acute exercise on mitochondrial fission/fusion mRNA and protein responses.

Author, Year [Reference]	Species/Model	Acute Exercise Stimulus	Summary Skeletal Muscle mRNA and/or Protein Responses	Evidence for Pro-Fission Responses	Evidence for Pro-Fusion Responses
Cartoni et al. 2005 [95]	Human (well trained cyclists); SAOS2 cell culture	45 min ∼80% VO2peak cycling	↑ *mfn1/2* mRNA (24 h post), via ERRα and PGC1α. ↔ MFN2 protein abundance 0–24 h post exercise	n/a	↑ *mfn1/2* mRNA (24 h post)
Ding et al. 2010 [104]	Rat	2.5 h ∼75% VO2peak treadmill running	↑ *mfn1* mRNA, but ↓ MFN1 protein (0–24 h post); ↑ *mfn2* mRNA 24 h post. ↑ *fis1* mRNA and FIS1 protein 0–24 h post	↑ *fis1* mRNA, ↑ FIS1 and ↓ MFN1 protein 0–24 h post	↑ *mfn1* mRNA (0–24 h post); ↑ *mfn2* mRNA 24 h post.
Perry et al. 2010 [96]	Human	1 h ∼90% VO2peak cycling, high intensity intervals	↔ MFN1/2 , FIS1 or DRP1 protein abundance 4 h post exercise	n/d	n/d
Picard et al. 2013 [105]	Mice	3 h voluntary running (∼1.8 km covered)	↑ intermitochondrial contacts; ↔ morphology or MFN2 and OPA1 protein abundance	n/d	↑ intermitochondrial contacts
Jamart et al. 2013 [106]	Mice	1.5 h low-intesntiy (~55% VO2 max) treadmill running	↑ DRP1 Ser616 phosphorylation, ↔ *dnm1l* mRNA, ↔ *mfn1/2* mRNA ~5 min post-exercise	↑ DRP1 Ser616 phosphorylation	n/d
Kitaoka et al. 2015 [97]	Rat	~1 h ‘resistance exercise’ electrical stimulation isometric contraction	↑ DRP1 Ser616 phosphorylation 0 h post exercise. ↔ DRP1, FIS1, MFN1/2, OPA1 protein 0–24 h post contraction	↑ DRP1 Ser616 phosphorylation 0 h post exercise	n/d
Kruse et al. 2017 [107]	Human (healthy controls and obese+T2DM)	1 h (70% VO2max) cycling	Healthy subjects 0 h post-exercise mRNA: ↑ *mfn2*, ↔ *opa1*, ↔ *dnm1l*, ↔ *fis1*; 3 h post-exercise mRNA: ↔ *mfn2*, ↔ *opa1*, ↓ *dnm1l*, ↔ *fis1*; Post-exercise protein content: ↑ MFN2 ↔ OPA1, DRP1; ↑ DRP1 Ser616 phosphorylation. Obese-T2DM subjects similar, except ↔ MFN2 post-ex protein content	↑ DRP1 Ser616 phosphorylation	↑ *mfn2* mRNA 0 h post; ↓ *dnm1l* mRNA 3 h post; ↑ MFN2 post-exercise protein content

↑, increased; ↓, decreased; ↔, no change; n/a, not assessed; n/d, not detected.

**Table 2:** Effects of long-term exercise training on mitochondrial fission/fusion mRNA and protein responses.

Author, Year [Reference]	Species/Model	Exercise Training Protocol	Summary Skeletal Muscle mRNA and/or Protein Responses	Evidence for Pro-Fission Responses	Evidence for Pro-Fusion Responses
Kirkwood et al. 1987 [94]	Rat	10 weeks, 5 day/week, 10–120 min/day moderate-high intensity treadmill running	↑ mitochondrial volume density % in vastus lateralis (VL) and soleus. ↓ mitochondrial surface:volume ratio in deep VL yet ↔ in superficial VL or soleus	n/d	↓ mitochondrial surface: volume
Perry et al. 2010 [96]	Human	2 weeks, 3–4 day/week, 1 h/day high intensity interval cycling exercise ∼90% VO2peak	↑ MFN1 , FIS1 and DRP1 protein; ↔ MFN2 protein after 2 week training	↑ FIS1 and DRP1 protein	↑ MFN1
Konopka et al. 2013 [99]	Human	12 weeks moderate intensity cycling exercise training	↑ MFN1/2 and FIS1 total protein	↑ FIS1 protein	↑ MFN1/2 protein
Feng et al. 2013 [100]	Rat	4 weeks treadmill training	↓ MFN2 protein in mitochondrial fraction, ↔ in total homogenate	↓ MFN2 in mito fraction	n/d
Iqbal et al. 2013 [57]	Rat	7 day, 3 h/day electrical stimulation	↑ thickness of the subsarcolemma (SS) mitochondrial layer by 58%. Intermyofibrillar (IMF) mitochondria 75% larger and more reticular. Protein in SS mitochondria: ↑ OPA1 (36%) and MFN2 (53%); ↓ DRP1 (13%), ↔ FIS1. Whole homogenate similar changes (therefore, not due to IMF)	n/d	↑ MFN2 and OPA1, ↓ DRP1 protein
Fealy et al. 2014 [108]	Human	12 weeks, 5 h/week, ~80% Hrmax	↔ DRP1 total protein, ↓ basal DRP1 Ser616 phosphorylation. ↑ *opa1* and *dnm1l* mRNA (basal)	↑ *dnm1l* mRNA	↑ *opa1* mRNA
Kitaoka et al. 2015 [97]	Rat	4 weeks ‘resistance exercise’ electrical stimulation isometric contraction	↑ OPA1 and MFN1/2 protein	n/d	↑ OPA1 and MFN1/2 protein
Marton et al. 2015 [101]	Rat	3 months treadmill running training	↑ FIS1, ↓ MFN1 protein content	↑ FIS1, ↓ MFN1 protein	
MacInnis et al. 2017 [98]	Human	2 weeks, 3 day/week single-leg cycling moderate and high intensity in either leg	↑ MFN2 protein in whole homogenate, but ↔ in type I or type II fibers analysed separately	n/a	↑ MFN2 protein
Wyckelsma et al. 2017 [103]	Human (older)	12 weeks, ~2 h/week cycling ~90% Hrmax	↓ MFN2 protein in type II fibers, but ↔ in type I or whole homogenate. ↔ MID49 in whole homogenate	↓ MFN2 protein in type II fibers	n/d

↑, increased; ↓, decreased; ↔, no change; n/a, not assessed; n/d, not detected.
